# IL28B Polymorphism Correlates with Active Hepatitis in Patients with HBeAg-Negative Chronic Hepatitis B

**DOI:** 10.1371/journal.pone.0058071

**Published:** 2013-02-28

**Authors:** I-Cheng Lee, Chen-Hao Lin, Yi-Hsiang Huang, Teh-Ia Huo, Chien-Wei Su, Ming-Chih Hou, Hui-Chun Huang, Kuei-Chuan Lee, Che-Chang Chan, Ming-Wei Lin, Han-Chieh Lin, Shou-Dong Lee

**Affiliations:** 1 Division of Gastroenterology, Department of Medicine, Taipei Veterans General Hospital, Taipei, Taiwan; 2 Endoscopy Center for Diagnosis and Treatment, Taipei Veterans General Hospital, Taipei, Taiwan; 3 Institute of Clinical Medicine, National Yang-Ming University School of Medicine, Taipei, Taiwan; 4 Institute of Pharmacology, National Yang-Ming University School of Medicine, Taipei, Taiwan; 5 Institute of Public Health, National Yang-Ming University School of Medicine, Taipei, Taiwan; 6 Department of Medicine, National Yang-Ming University Hospital, I-Lan, Taiwan; 7 Cheng Hsin General Hospital, Taipei, Taiwan; Kaohsiung Medical University Hospital, Kaohsiung Medical University, Taiwan

## Abstract

**Background & Aims:**

The clinical relevance of single nucleotide polymorphisms (SNPs) near the *IL28B* gene is controversial in patients with hepatitis B virus (HBV) infection. This study aimed to investigate the role of viral and host factors, including *IL28B* genotypes, in the natural course of chronic hepatitis B (CHB).

**Methods:**

The study enrolled consecutive 115 treatment-naive CHB patients. HBV viral loads, genotypes, precore and basal core promotor mutations, serum hepatitis B surface antigen (HBsAg) and interferon-gamma inducible protein 10 (IP-10) levels as well as four SNPs of *IL28B* were determined. Serial alanine transaminase (ALT) levels in the previous one year before enrollment at an interval of three months were recorded. Factors associated with active hepatitis, defined as persistent ALT >2× upper limit of normal (ULN) or a peak ALT level >5× ULN, were evaluated.

**Results:**

The prevalence of rs8105790 TT, rs12979860 CC, rs8099917 TT, and rs10853728 CC genotypes were 88.3%, 87.4%, 88.4% and 70.9%, respectively. In HBeAg-positive patients (n = 48), HBV viral load correlated with active hepatitis, while in HBeAg-negative patients (n = 67), rs10853728 CC genotype (p = 0.032) and a trend of higher IP-10 levels (p = 0.092) were associated with active hepatitis. In multivariate analysis, high viral load (HBV DNA >10^8^ IU/mL, p = 0.042, odds ratio = 3.946) was significantly associated with HBeAg-positive hepatitis, whereas rs10853728 CC genotype (p = 0.019, odds ratio = 3.927) was the only independent factor associated with active hepatitis in HBeAg-negative population.

**Conclusions:**

HBV viral load and IL28B rs10853728 CC genotype correlated with hepatitis activity in HBeAg-positive and HBeAg-negative CHB, respectively. Both viral and host factors play roles in disease activity during different phases of CHB.

## Introduction

Hepatitis B virus (HBV) infection is an important cause of chronic liver disease globally, with an estimated 350 million carriers worldwide [1]. Patients with chronic hepatitis B (CHB) are at increased risks of developing cirrhosis, hepatic decompensation, and hepatocellular carcinoma (HCC), either of which can lead to a liver-related death [2]. Chronic HBV infection is a dynamic state of the interactions between virus and host immune response, and the natural course varies greatly among different individuals, while some patients had frequent hepatitis flares with more rapid progression of liver disease, others were at inactive carrier state with a relative benign prognosis [3].

Several host and viral factors have been reported to be associated with the natural course of CHB [4]. Genotypes, basal core promoter (BCP) mutations and viral loads of HBV may influence the progression of HBV-related liver disease [4], [5], [6]. Similarly, age, sex, host immune status and metabolic factors of the host also correlate with disease progression in CHB [4], [7]. Patients with higher baseline alanine transaminase (ALT) levels had a better response to interferon (IFN) therapy [8], indicating that host immune response plays an important role in eliminating HBV. Currently, the genetic determinants of host immune responses to HBV infection remain unclear.

Genome-wide association studies have shown that single-nucleotide polymorphisms (SNPs) at or near the *interleukin 28B* gene (*IL28B*) region on chromosome 19, which encodes interferon-lamda 3 (IFN-λ3), are associated with spontaneous hepatitis C virus (HCV) clearance and sustained virological response (SVR) in patients with chronic hepatitis C (CHC) treated with pegylated-interferon (PEG-IFN) and ribavirin [9], [10], [11], [12], [13]. Furthermore, a recent study showed that patients with lower serum levels of interferon gamma-inducible protein 10 (IP-10) in combination with favorable *IL28B* genotypes had higher chance of spontaneous HCV clearance [14]. Whether *IL28B* polymorphisms have influence on host immune response to HBV infection is unknown. It is possible that genetic variations at *IL28B* region determine the host susceptibility to HBV infection and influence the progression of liver disease.

Currently, whether IL28B genotypes are associated with the natural course of HBV infection and hepatitis activity in CHB is not fully understood. This study aimed to investigate the role of viral and host factors, including *IL28B* genotypes, in the natural course of chronic hepatitis B (CHB).

## Materials and Methods

### Patients

From April 2009 to July 2011, consecutive 115 treatment-naïve CHB patients who were willing to participate in this study at the Taipei Veterans General Hospital were enrolled. All patients were positive for serum HBsAg for more than 6 months and had documented elevation of serum ALT levels [>40 U/L, 1× upper limit of normal (ULN)] with HBV DNA >2,000 IU/mL [15], [16], [17]. All patients had been regularly followed with three-month interval for at least one year. Patients were negative for any of the following points: (1) coinfection with HCV, hepatitis D virus, or human immunodeficiency virus, (2) alcoholic liver disease, (3) suspected autoimmune disease with antinuclear antibody (ANA) titer ≥1∶160, positive test for anti-smooth muscle antibody or anti-mitochondrial antibody, (4) use of hepatotoxic drug or Chinese herb, and (5) radiological evidence of cirrhosis or HCC (i.e., abdominal sonogram, computed tomography scan, or magnetic resonance imaging scans). This study was approved by the Institutional Review Board, Taipei Veterans General Hospital, which complied with standards of the Declaration of Helsinki and current ethical guidelines. All patients provided written informed consents for participation of the study and for use of genetic material for this study.

Peripheral blood samples were obtained from all patients for serological, virological tests and *IL28B* genotypes analyses. Serial ALT levels in the previous one year before enrollment at an interval of three months were recorded. Active hepatitis was defined as persistent ALT >2× ULN or a peak ALT level >5× ULN. Patients who did not fulfill the criteria were defined as mild hepatitis.

### Liver biochemistry and viral serology tests

Serum biochemical studies were performed using a systemic multi-autoanalyzer (Technicon SMAC, Technicon Instruments Corp., Tarrytown, NY). The serum samples were tested for the presence of HBeAg and anti-HBe antibody using radio-immunoassay (Abott Laboratories, North Chicago, IL), while a Cobas Amplicor HBV monitor determined HBV DNA (detection limit of 12 IU/mL).

### *IL28B* genotyping

Four SNPs of *IL28B* including rs8105790, rs12979860, rs8099917 and rs10853728 were chosen according to previous reports [9], [10], [11], [12], [18], [19], [20]. The genotype of rs12979860 was tested using TaqMan custom-designed rs12979860 probes (Applied Biosystems, Foster City, CA; forward primer GCCTGTCGTGTACTGAACCA, reverse primer GCGCGGAGTGCAATTCAAC, and the probes TGGTTCGCGCCTTC [VIC] and CTGGTTCACGCCTTC [FAM], respectively) [21]. The genotypes of the rs8105790, rs8099917 and rs10853728 were determined with the ABI TaqMan SNP genotyping assays (Applied Biosystems) and with predesigned commercial genotyping assays (ABI assay C__43813808_10, C__11710096_10, C__11710090_10). Briefly, PCR primers and two allelic-specific probes was designed to detect a specific SNP target. The PCR reactions were performed in 96-well microplates with ABI 7900 real-time PCR (Applied Biosystems).

### Detection, genotyping and sequencing of HBV DNA

Genotyping of HBV was performed by PCR restriction fragment length polymorphism (PCR-RFLP) of the surface gene of HBV [6], [22]. Briefly, DNA was extracted from serum, and the fragment of the HBV genome between nucleotide position 120 and 604 was amplified by semi-nested PCR. The PCR products were subsequently treated with restriction enzymes. After incubation, the samples were run on a 4% agarose gel and stained by ethidium bromide. To confirm the correct genotyping, direct sequencing from the PCR products was done.

To detect precore G1896A and basal core promoter (BCP) A1762T/G1764A mutations, sequencing of the core region of HBV DNA was performed in all patients. Semi-nested PCR was performed by using a pair of primers: internal primers 1653F (5′-CATAAGAGGACTCTTGGACT-3′, position 1653-1672) and 1974R (5′-GGAAAGAAGTCAGAAGGC-3′, position 1974–1957); external primers: 1623F (5′-TCGCATGGAGACCACCGTCT-3′, position 1623–1640) and 2076R (5′-ATAGCTTGCCTGAGTGC-3′, position 2076–2060) as previously described [6], [22].

The PCR products were then subjected to the dye-terminator cycle sequencing reaction using specific primers according to the standard protocol provided by the manufacturer (Dye terminator cycle sequencing core kit no. 402117, Perkin Elmer Cetus Corp., Norwalk, CT). To avoid false positive results, instructions to prevent cross contaminations were strictly followed.

### Serum HBsAg quantification

HBsAg levels were quantified using the Elecsys HBsAg II assay (Roche Diagnostics GmbH, Mannheim, Germany) according to the manufacturer's instructions [23]. The detection limit of the Elecsys HBsAg II assay was 0.05 IU/mL.

### Enzyme-linked sorbent assay (ELISA) for IP-10 detection

The concentrations of interferon gamma-inducible protein 10 (IP-10) in serum were tested by commercialized human IP-10 ELISA development kit (PeproTech, Rocky Hill, NJ) in this study. The procedure followed the instruction provided by manufactures. The lower detection limit was 8 pg/mL for IP-10.

### Statistical analyses

Linkage disequilibrium (LD) between the four candidate SNP loci was assessed and haplotype blocks were constructed using Haploview 4.2 [24]. All statistical analyses were performed using the Statistical Package for Social Sciences (SPSS 17.0 for Windows, SPSS Inc, Chicago, IL). Values were expressed as median (ranges) or as mean ± standard deviation when appropriate. Pearson chi-square analysis or Fisher exact test was used to compare categorical variables, while the Student t test or Mann-Whitney U test was used to compare continuous variables. Variables with p<0.1 were analyzed by multivariate logistic regression analysis to identify independent variables for predicting active hepatitis. A 2-tailed p value <0.05 was considered statistically significant.

## Results

### Patient characteristics

The baseline characteristics of the 115 CHB patients are summarized in Table 1. Patients were predominantly male (73%) and HBeAg-negative (58.3%). Sixty-seven patients (58.3%) had active hepatitis. HBV genotypes were determined in all patients, with 3.5% genotype A, 64.3% genotypes B and 32.2% genotype C. Compared with HBeAg-positive patients, HBeAg-negative patients were older, had significantly lower HBV DNA, HBsAg and IP-10 levels (Table 1). About three-fourth of HBeAg-negative patients were infected with genotype B HBV, whereas 45% of HBeAg-positive patients were infected with genotype C HBV (p = 0.006).

**Table 1:** Baseline characteristics of the 115 chronic hepatitis B patients.

	All patients n = 115	HbeAg-positive n = 48 (41.7%)	HbeAg-negative n = 67 (58.3%)	p value
Age (years)	45±13	38±11	50±12	<0.01
Male sex, n (%)	84 (73)	31 (64.6)	53 (79.1)	0.13
Active hepatitis, n (%)*	67 (58.3)	31 (64.6)	36 (53.7)	0.33
Genotype A/B/C, n (%)	4/74/37 (3.5/64.3/32.2)	0/26/22 (0/54.2/45.8)	4/48/15 (6/71.6/22.4)	<0.01
Basal core promoter mutation, n (%)	39 (34.2)	15 (31.9)	24 (35.8)	0.82
Precore mutation, n (%)	55 (47.8)	15 (31.3)	40 (59.7)	<0.01
HBV DNA (Log_10_ IU/mL)	6.52±1.36	7.39±0.98	5.90±1.26	<0.01
HBsAg (Log_10_ IU/mL)	3.49 (1.39–5.46)	4.00 (2.05–5.46)	3.14 (1.39–4.87)	<0.01
IP-10 (pg/mL)	78.6 (13.9–2078)	97.3 (19.1–2078)	65.4 (13.9–1301.7)	<0.01
ALT (U/L)	211±169	235±161	194±174	0.06
AST (U/L)	120±117	130±121	112±114	0.24
*IL28B* polymorphisms				
rs8105790 TT/CT + CC, n (%)	98/13 (88.3/11.7)	39/7 (84.8/15.2)	59/6 (90.8/9.2)	0.51
rs12979860 CC/CT + TT, n (%)	97/14 (87.4/12.6)	39/7 (84.8/15.2)	58/7 (89.2/10.8)	0.69
rs8099917 TT/GT + GG, n (%)	99/13 (88.4/11.6)	39/7 (84.8/15.2)	60/6 (90.9/9.1)	0.49
rs10853728 CC/CG + GG, n (%)	78/32 (70.9/29.1)	33/13 (71.7/28.3)	45/19 (70.3/29.7)	1.00

Abbreviations: HBeAg, hepatitis B e antigen; HBsAg, hepatitis B surface antigen; IP-10, interferon-gamma inducible protein 10; ALT, alanine transaminase; AST, aspartate transaminase.

* Active hepatitis was defined as persistent ALT >2× upper limit of normal (ULN) or a peak ALT level >5× ULN.

Thirty-nine patients (34.2%) had BCP mutations and 55 (47.8%) had precore mutations. The prevalence of BCP mutations was comparable between HBeAg-positive and HBeAg-negative patients, whereas the prevalence of precore mutations was significantly higher in HBeAg-negative patients (Table 1). Genotype C HBV had a significantly higher prevalence of BCP mutations (26% in genotype B vs. 51.4% in genotype C, p = 0.015), whereas genotype B HBV was precore mutations predominant (54.1% in genotype B vs. 32.4% in genotype C, p = 0.051). In HBeAg-positive patients, the prevalence of BCP mutations were 16% (4/25) in genotype B and 50% (11/22) in genotype C (p = 0.029), and the prevalence of precore mutations were 38.5% (10/26) in genotype B and 22.7% (5/22) in genotype C (p = 0.390). In HBeAg-negative patients, the prevalence of BCP mutations were 25% (1/4) in genotype A, 31.3% (15/48) in genotype B and 53.3% (8/15) in genotype C (p = 0.277), and the prevalence of precore mutations were 75% (3/4) in genotype A, 62.5% (30/48) in genotype B and 46.7% (7/15) in genotype C (p = 0.448).

### Distribution of *IL28B* Genotypes

**Figure 1:**
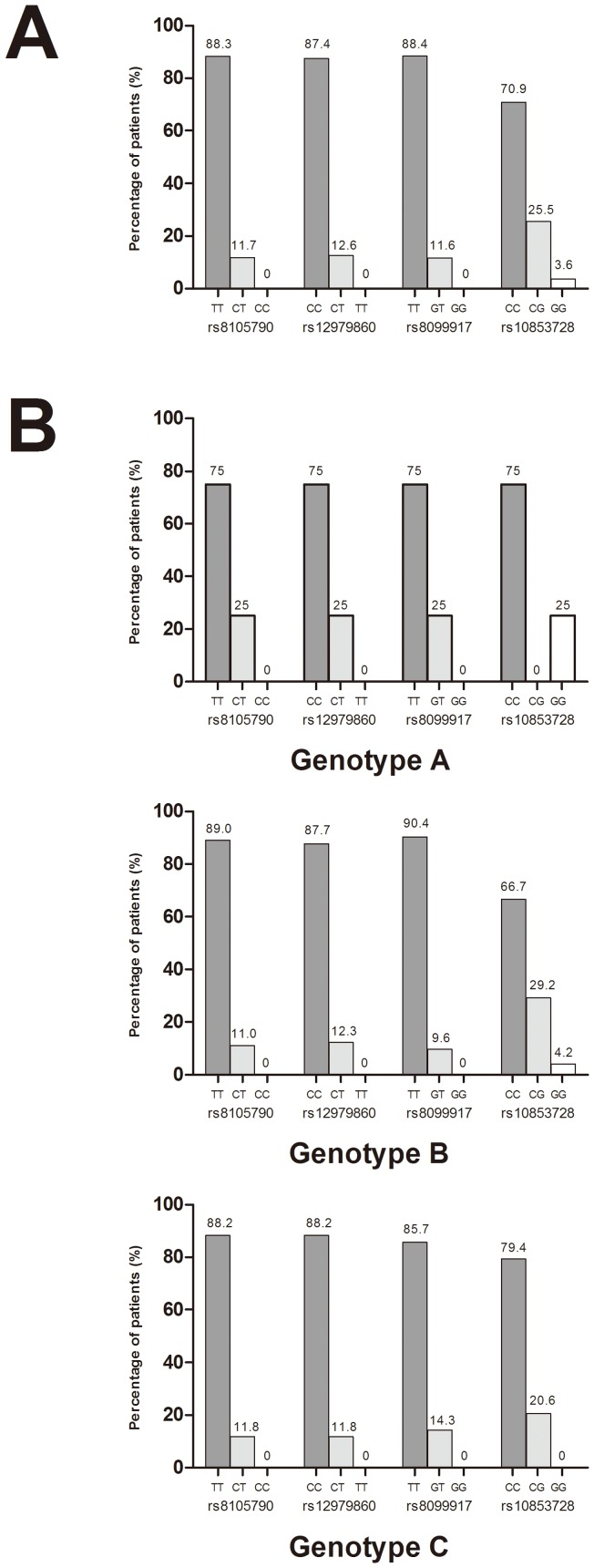
Distributions of *IL28B* polymorphisms. (A) Distributions of rs8105790, rs12979860, rs8099917 and rs10853728 genotypes in overall patients. (B) Distributions of rs8105790, rs12979860, rs8099917 and rs10853728 genotypes in patients infected with HBV genotypes A, B and C.

**Figure 2:**
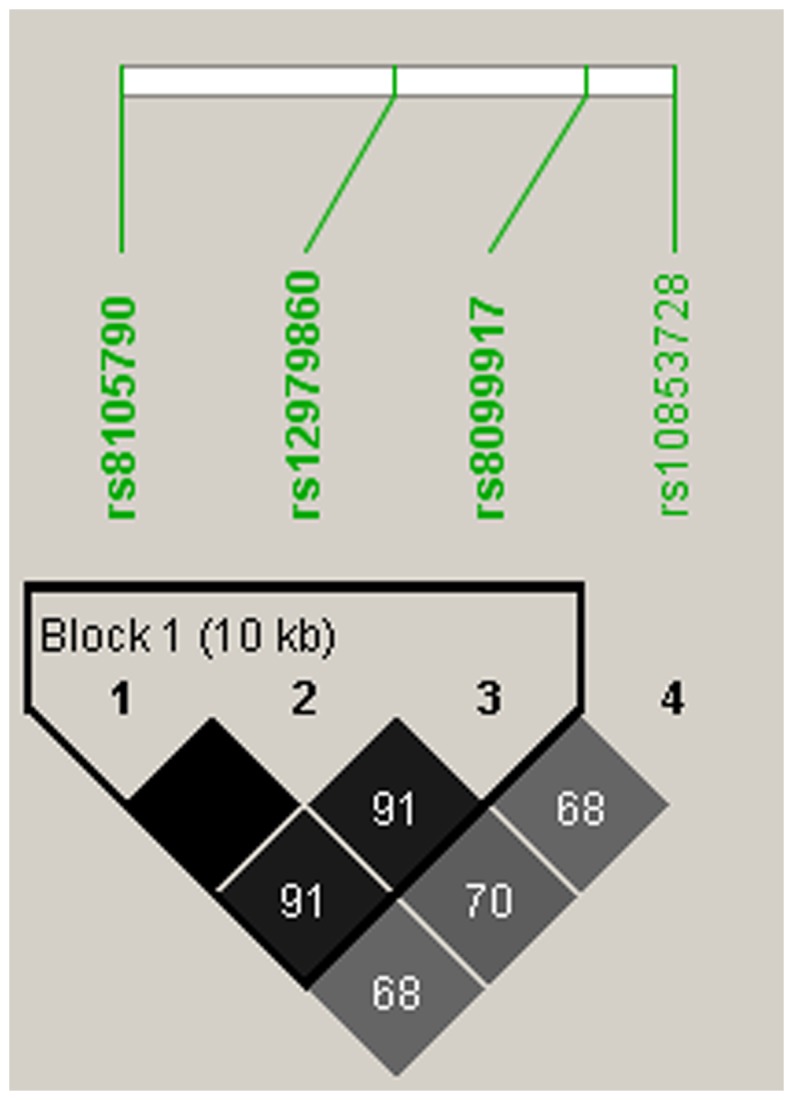
Pairwise linkage disequilibrium (LD) patterns of the four single nucleotide polymorphisms through *IL28B* regions. Pairwise r^2^ was used for analyzing the correlation.

Among the 115 CHB patients, 112 (97.4%) had determined rs8099917 genotype, 111 (96.5%) had determined rs8105790 and rs12979860 genotypes, and 110 (95.7%) had determined rs10853728 genotype. The distributions of the four SNPs of *IL28B* in overall patients and in patients infected with HBV genotypes A, B and C are shown in Figure 1. The prevalence of the major genotypes rs8105790 TT, rs12979860 CC, rs8099917 TT, and rs10853728 CC were 88.3%, 87.4%, 88.4% and 70.9%, respectively. The distributions of the four SNPs were not significantly different among patients infected with HBV genotypes A, B or C (p = 0.749, 0.787, 0.581 and 0.383 for rs8105790, rs12979860, rs8099917 and rs10853728, respectively). Among the four SNPs, rs8105790 and rs12979860 were highly linked, with only 1 (0.9%) patient classified discordantly (*r* ^2^ = 0.78, Figure 2). The rs10853728 was not so closely linked with the other 3 SNPs (Figure 2). There were no significant correlations between the HBeAg status and the four SNPs of *IL28B* (Table 1).

### Factors associated with active hepatitis in CHB patients

In overall patients, serum IP-10 levels were significantly higher in patients with active hepatitis (p = 0.021, Table 2), whereas age, sex, HBeAg status, HBV genotypes, BCP or precore mutations, viral loads, HBsAg levels, or the four SNPs of *IL28B* were not correlated with active hepatitis.

**Table 2:** Characteristics of the chronic hepatitis B patients stratified by hepatitis activity*.

	All patients (n = 115)			HBeAg-positive (n = 48)			HBeAg-negative (n = 67)		
	Mild hepatitis n = 48 (41.7%)	Active hepatitis n = 67 (58.3%)	p value	Mild hepatitis n = 17 (35.4%)	Active hepatitis n = 31 (64.6%)	p value	Mild hepatitis n = 31 (46.3%)	Active hepatitis n = 36 (53.7%)	p value
Age (years)	46±13	44±14	0.38	40±13	36±11	0.39	50±11	51±12	0.72
Male sex, n (%)	34 (70.8)	50 (74.6)	0.81	8 (41.7)	23 (74.2)	0.12	26 (83.9)	27 (75)	0.56
Genotype A/B/C, n (%)	1/31/16 (2.1/64.6/33.3)	3/43/21 (4.5/64.2/31.3)	0.77	0/10/7 (0/58.8/41.2)	0/16/15 (0/51.6/48.4)	0.86	1/21/9 (3.2/67.7/29.0)	3/27/6 (8.3/75/16.7)	0.36
HBV DNA (Log_10_ IU/mL)	6.38±1.29	6.62±1.42	0.16	6.99±1.15	7.61±0.80	0.02	6.04±1.25	5.78±1.28	0.49
HBsAg (Log_10_ IU/mL)	3.46 (1.86–5.27)	3.50 (1.39–5.46)	0.63	3.59 (2.05–5.27)	4.11 (2.12–5.46)	0.58	3.30 (1.86–4.82)	3.05 (1.39–4.87)	0.55
Basal core promoter mutation, n (%)	17 (35.4)	22 (33.3)	0.98	7 (41.2)	8 (26.7)	0.48	10 (32.3)	14 (38.9)	0.76
Precore mutation, n (%)	20 (41.7)	35 (52.2)	0.35	4 (23.5)	11 (35.5)	0.60	16 (51.6)	24 (66.7)	0.32
IP-10 (pg/mL)	67.6 (19–832.9)	87.5 (13.9–2078)	0.02	93.2 (19.1–198.3)	97.3 (37.1–2078)	0.27	59.8 (19–832.9)	78.6 (13.9–1301.7)	0.09
ALT (U/L)	108±40	284±188	<0.01	111±46	303±161	<0.01	109±38	267±209	<0.01
AST (U/L)	65±25	158±139	<0.01	74±33	161±140	<0.01	61±17	156±141	<0.01
*IL28B* polymorphisms									
rs8105790			1.00			1.00			1.00
TT	40 (40.8%)	58 (59.2%)		14 (35.9%)	25 (64.1%)		26 (44.1%)	33 (55.9%)	
CT + CC	5 (38.5%)	8 (61.5%)		2 (28.6%)	5 (71.4%)		3 (50%)	3 (50%)	
rs12979860			1.00			1.00			0.69
CC	39 (40.2%)	58 (59.2%)		14 (35.9%)	25 (64.1%)		25 (43.1%)	33 (56.9%)	
CT + TT	6 (42.9%)	8 (57.1%)		2 (28.6%)	5 (71.4%)		4 (57.1%)	33 (56.9%)	
rs8099917			0.62			0.39			1.00
TT	42 (42.4%)	57 (57.6%)		15 (38.5%)	24 (61.5%)		27 (45%)	33 (55%)	
GT + GG	4 (30.8%)	9 (69.2%)		1 (14.3%)	6 (85.7%)		3 (50%)	3 (50%)	
rs10853728			0.15			1.00			0.03
CC	28 (35.9%)	50 (64.1%)		12 (36.4%)	21 (63.6%)		16 (35.6%)	29 (64.4%)	
CG + GG	17 (53.1%)	15 (46.9%)		4 (30.8%)	9 (69.2%)		13 (68.4%)	6 (31.6%)	

* Active hepatitis was defined as persistent ALT >2× ULN or a peak ALT level >5× ULN, while patients who did not fulfill the criteria were defined as mild hepatitis.

We further stratified the CHB patients by HBeAg status. Factors associated with active hepatitis in HBeAg-positive and HBeAg-negative patients were shown in Table 2. In HBeAg-positive CHB patients, those with active hepatitis had significantly higher serum HBV viral load, ALT and AST levels. In contrast, serum levels of HBsAg, IP-10, HBV genotypes, precore/BCP mutations and the four SNPs of *IL28B* were not associated with hepatitis activity.

**Figure 3:**
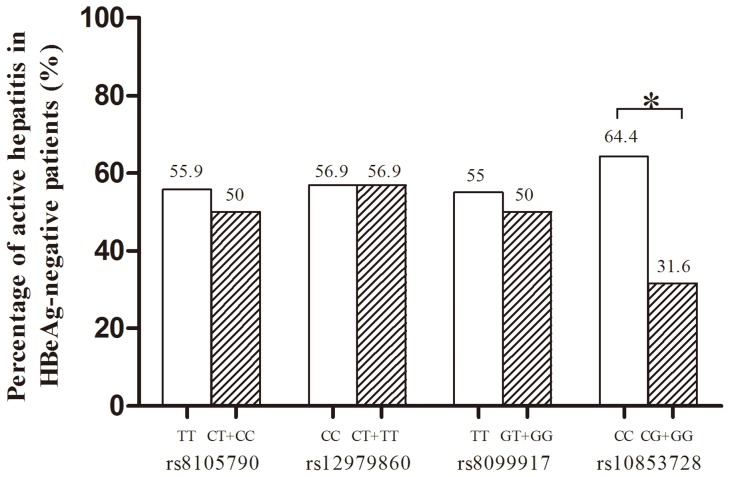
Association of rs8105790, rs12979860, rs8099917 and rs10853728 genotypes with hepatitis activity. *p = 0.032.

In HBeAg-negative patients, those with active hepatitis had significantly higher serum ALT and AST levels, a trend of higher IP-10 levels (p = 0.092), and patients with rs10853728 CC genotype were more susceptible to active hepatitis (p = 0.032, Figure 3). In contrast, rs8105790, rs12979860, rs8099917 polymorphisms were not associated with active hepatitis. The HBV genotypes, precore/BCP mutations and HBV viral load were not associated with active hepatitis in HBeAg-negative patients.

In multivariate analysis, high viral load (HBV DNA >10^8^ IU/mL, p = 0.042, odds ratio = 3.946) was the significant factor associated with active hepatitis in HBeAg-positive patients, while rs10853728 CC genotype (p = 0.019, odds ratio = 3.927) was the only independent factor associated with active hepatitis in HBeAg-negative population (Table 3). Among patients with lower viral loads (HBV DNA <5×10^7^ IU/mL), not only rs10853728 CC genotype (p = 0.041, odds ratio = 2.991) but also IP-10>75 pg/mL (p = 0.022, odds ratio = 3.062) were independently associated with active hepatitis.

**Table 3:** Factors associated with active hepatitis in HBeAg-positive and HBeAg -negative patients, by multivariate analysis.

Variables	p value	Odds ratio	95% confidence interval
HBeAg-positive patients			
HBV DNA >10^8^ IU/mL	0.042	3.946	1.049–14.851
HBeAg-negative patients			
rs10853728 CC genotype	0.019	3.927	1.251–12.326

## Discussion

Consistent with previous reports, serum HBV viral load and HBsAg levels were lower in HBeAg-negative patients as compared with HBeAg-positive cases [25], [26], [27]. We also observed that serum IP-10 levels were higher in HBeAg-positive patients. There was a significant correlation between IP-10 and active hepatitis, especially in patients with lower viral loads, but IP-10 was not associated with active hepatitis in HBeAg-positive patients with high viral load. Although the role of IP-10 in the natural history of CHB is unclear, recent studies showed that IP-10 plays a major role in the immune activation during hepatic failure in CHB, and a higher IP-10 level might be associated with HBsAg seroclearance during antiviral therapy [28], [29]. Both findings support that higher serum IP-10 levels in patients with active hepatitis represents stronger immune response to clear the virus. The impact of IP-10 on active hepatitis in HBeAg-positive population might be masked by viral factors.

HBV genotypes also play an important role in the natural history of CHB. Consistent with previous observations that patients with genotypes B HBV have earlier HBeAg seroconversion than genotype C infection [30], [31], our data showed genotype B HBV was predominant in HBeAg-negative patients whose age were older. A similar distribution of the four *IL28B* SNPs in patients infected with different HBV genotypes suggests that *IL28B* polymorphisms may not influence the susceptibility of infection to varied genotypes of HBV.

It has been recognized that BCP mutation is associated with genotype C HBV and precore mutation is associated with genotype B HBV [6], [32]. In the current study, we also observed this association. BCP mutation has been shown to correlate with progression of liver fibrosis and development of HCC, whereas precore mutation was associated with reduced risk of HCC [5], [33]. It is interesting that whether these mutations contribute to active hepatic necroinflammation. However, our results showed neither BCP nor precore mutations were related to active hepatitis in both HBeAg-positive and HBeAg-negative patients. The impact of the mutations on hepatic necroinflammation may not be revealed by this one-year observational study.

In our patients, the prevalence of IL28B SNPs rs8105790 TT, rs12979860 CC and rs8099917 TT genotypes were approximately 88%, while 70.9% of patients had rs10853728 CC genotype. The distributions of these four SNPs in our study were similar to that reported in CHC patients in Taiwan [18], [19]. Based on linkage disequilibrium finding, rs8105790 TT, rs12979860 CC and rs8099917 TT alleles are closely linked. Therefore, either one of the three SNPs genotype can represent the other two for Taiwanese.

In this study, we demonstrated that HBV viral load correlated with active hepatitis in HBeAg-positive patients, while *IL28B* rs10853728 CC genotype was associated with active hepatitis in HBeAg-negative patients (Figure 3). The result indicates that viral factor is the major determinant of hepatic inflammation in HBeAg-positive patients, while host factor, the *IL28B* polymorphisms, may contribute to hepatic inflammation in HBeAg-negative cases, as a consequence of host immune response to HBV. Our enrolled HBeAg-positive patients with elevated liver enzymes and HBV viral load >2,000 IU/ml represents immune clearance phase in the natural history of HBV infection [3]. These patients tend to have higher HBV viral loads and therefore host factor might be overwhelmed by viral factor being responsible for disease activity. Indeed, in patients with lower viral loads, i.e. HBV DNA <5×10^7^ IU/mL, the role of rs10853728 CC genotype in hepatitis activity become predominant. On the contrary, in HBeAg-negative patients with a relatively lower viral load, the significance of rs10853728 CC genotype will not be masked by viral load and is the only factor associated with active hepatitis. Although rs10853728 genotype was not emphasized in studies of hepatitis C, a recent study showed that rs10853728 CC was significantly associated spontaneous HCV clearance in Chinese population [20]. Our findings suggest that certain *IL28B* genotype might relate to host immune response against viral infection. We defined “active hepatitis” as those cases might have stronger host immune response against HBV infection. A favorable *IL28B* genotype in HCV infection is associated with spontaneous and IFN-induced viral clearance, indicating that *IL28B* genes might be responsible for triggering immune response against HCV during IFN treatment. A recent study by Sonneveld MJ, et al also discovers that *IL28B* polymorphism is associated with HBeAg seroconversion in HBeAg-positive CHB under IFN treatment [34]. Although the evidence is not clear enough, this *IL28B* polymorphism might associate with a higher ALT at baseline in those patients, which is a well-known predictor of HBeAg seroconversion for HBeAg-positive patients [4], [15], [16], [17]. In our current study, rs10853728 CC genotype correlates with a higher hepatitis activity, suggesting that the *IL28B* genotypes may also trigger intrinsic host antiviral immune response against HBV infection in HBeAg-negative patients. Although active hepatitis may induce liver disease progression, a higher ALT levels may also represent a stronger host immune response to HBV. Therefore, our finding is not descrepant with previous findings in patients with hepatitis C. The long-term outcome of IL28B related active hepatitis is still unclear and needs further extended observation.

Currently, the association between *IL28B* polymorphisms and the outcomes of CHB remains controversial. Some studies showed that *IL28B* genotypes were not associated with recovery from HBV infection, clearance of HBeAg and HBsAg, hepatitis activity and liver cirrhosis [35], [36], while other studies showed significant correlations between *IL28B* genotypes and HBV viral load, hepatic inflammation and risk of HCC [37], [38]. The discrepancies between these studies may result from racial difference, and the enrolled patients were in different phases of CHB.

Two recent studies showed that *IL28B* SNP may predict response to PEG-IFN in HBeAg-positive CHB patients, indicating that host factor is important for the induction of an immune response during antiviral therapy in CHB [34], [39]. Recently, Lampertico et al. also showed that *IL28B* polymorphism was associated with interferon-related HBsAg seroclearance in genotype D HBeAg-negative patients [40]. Although these studies provide promising role of *IL28B* polymorphisms in CHB, rs10853728 genotype was not tested in previous HBV studies. *IL28B* rs10853728 genotype might also have clinical implication in the treatment of HBeAg-negative CHB in the future.

There is evidence that the gene product of *IL28B*, IFN- λ3, up-regulates interferon-stimulated genes, which may regulate many of the innate cellular defenses against viral infection [41]. In addition, *IL28B* is also a regulator of the adaptive immune response by regulating the number and function of CD8^+^ T cells [42]. The association of rs10853728 CC genotype with active hepatitis in HBeAg-negative patients implies that *IL28B* gene polymorphism may dominate host immune activity in the course of CHB.

The correlation between *IL28B* SNPs and IP-10 secretion during viral infection are not yet established. Previous studies in CHC showed that patients with rs12979860 CC genotype had lower IP-10 levels [14], [43]. Our data showed that there was no correlation between *IL28B* SNPs and IP-10 levels, but in patients with low viral load (HBV DNA <5×10^7^ IU/mL), rs12979860 CC genotype was associated with higher IP-10 levels (mean 148.6 pg/mL in CC type vs. 128.9 pg/mL in non-CC type, p = 0.021,). In contrast, rs10853728 CC genotype was not associated with IP-10 level. The scenario of IP-10 in response to viral infection might be different between HBV and HCV infection. Our data suggests that certain IL28B gene may relate to the susceptibility of IP-10 secretion during chronic HBV infection.

This study has some limitations. Our studied population was not large enough, which may lead to potential type I error caused by multiple comparisons. A multicenter study to include more patients to confirm the role of IL28B in the natural history of CHB is necessary in the future. Our studied population did not include inactive HBV carriers. Therefore, whether genetic variations in *IL28B* contribute to disease remission in inactive HBV carriers with persistently normal ALT could not be elucidated in this study. Our enrolled patients did not receive liver biopsy, which is currently the gold standard to evaluate hepatic necroinflammation. However, current guidelines do not suggest routine liver biopsy before antiviral therapy in CHB patients, especially those with obviously active CHB, i.e. ALT above 2 times ULN and HBV DNA >2000 IU/mL [15], [16]. Furthermore, regarding the fluctuating nature of CHB, serial ALT levels may also reflect the long term course of hepatic necroinflammation.

In conclusion, HBV viral load is related to active hepatitis in HBeAg-positive CHB, while *IL28B* rs10853728 CC genotype is associated with active hepatitis in HBeAg-negative CHB. Both viral and host factors play roles in disease activity during different phases of CHB. The role of rs10853728 genotype in predicting long-term outcome and antiviral response for HBeAg-negative CHB warrants future study.
